# Supplementary material for: Machine learning-based meta-analysis reveals gut microbiome alterations associated with Parkinson’s disease
Source: Nat Commun. 2025 May 7;16:4227. doi: 10.1038/s41467-025-56829-3 (PMC12059030; doi:10.1038/s41467-025-56829-3)

Supplementary figures

Supplementary Fig. 1 | Sample dissimilarities between studies are significantly higher than those within studies. *For all 16S and SMG data, Bray-Curtis dissimilarities were divided into two groups: "Within Studies" and "Between Studies". The distributions of the dissimilarities are depicted using boxplots, in which 50% of the data (25th to 75th percentile) are within the limits of the box and the thick vertical line within it indicates the data's median (50th percentile). Outliers have not been plotted. Differences in the distributions of the dissimilarities between the two groups were then tested using a two-sample t-test (16S: t = 402.6, df = 9598588, *** p-value < 2.2e^{-16}, effect size = 0.64, 95% confidence interval = 0.63 - 0.64; SMG: t = 300.7, df = 1457191, *** p-value < 2.2e^{-16}, effect size = 0.62, 95% confidence interval = 0.62 - 0.62)*

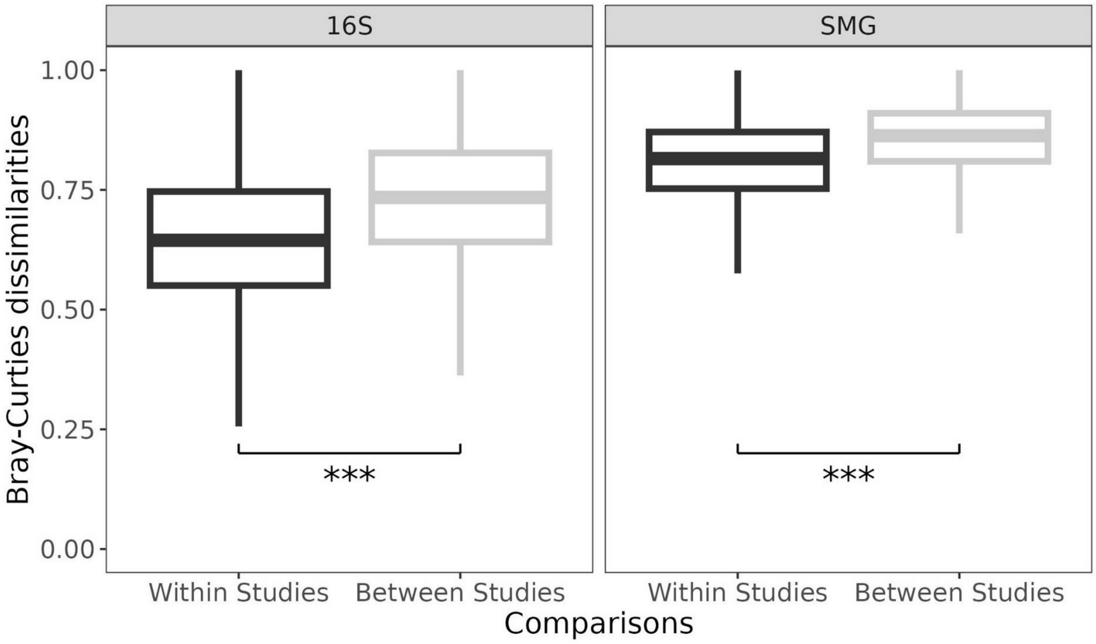

Supplementary Fig. 2 | AUCs across ML algorithms, normalizations, and taxa filtration thresholds. *Results refer to ML models built using all 16S amplicon datasets included in our meta-analysis (n = 17 datasets). Distribution of AUCs is depicted using boxplots, in which 50% of the data (25th to 75th percentile) are within the limits of the box and the thick vertical line within it indicates the data's median (50th percentile).*

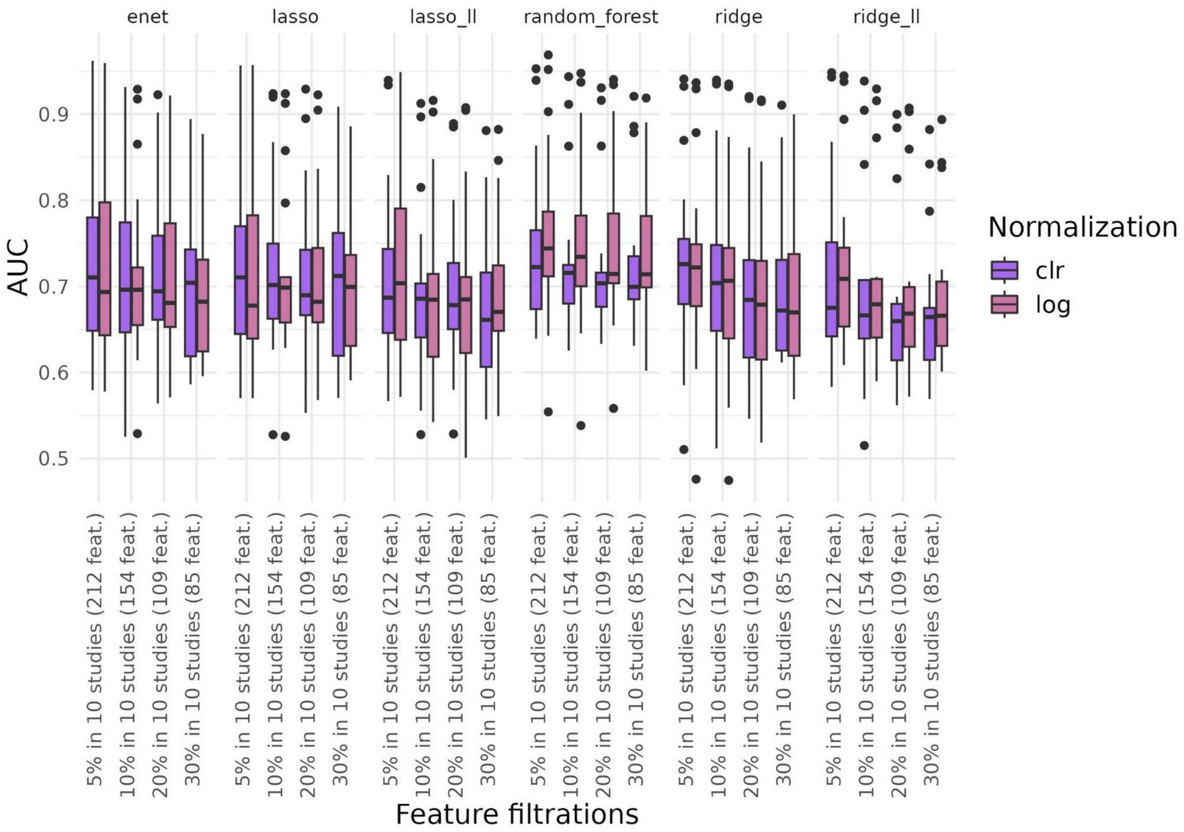

Supplementary Fig. 3 | AUCs across ML algorithms, normalizations, and taxa filtration parameters. *Results refer to ML models built using all shotgun metagenomics datasets included in our meta-analysis (n = 7 datasets). Distribution of AUCs is depicted using boxplots, in which 50% of the data (25th to 75th percentile) are within the limits of the box and the thick vertical line within it indicates the data's median (50th percentile).*

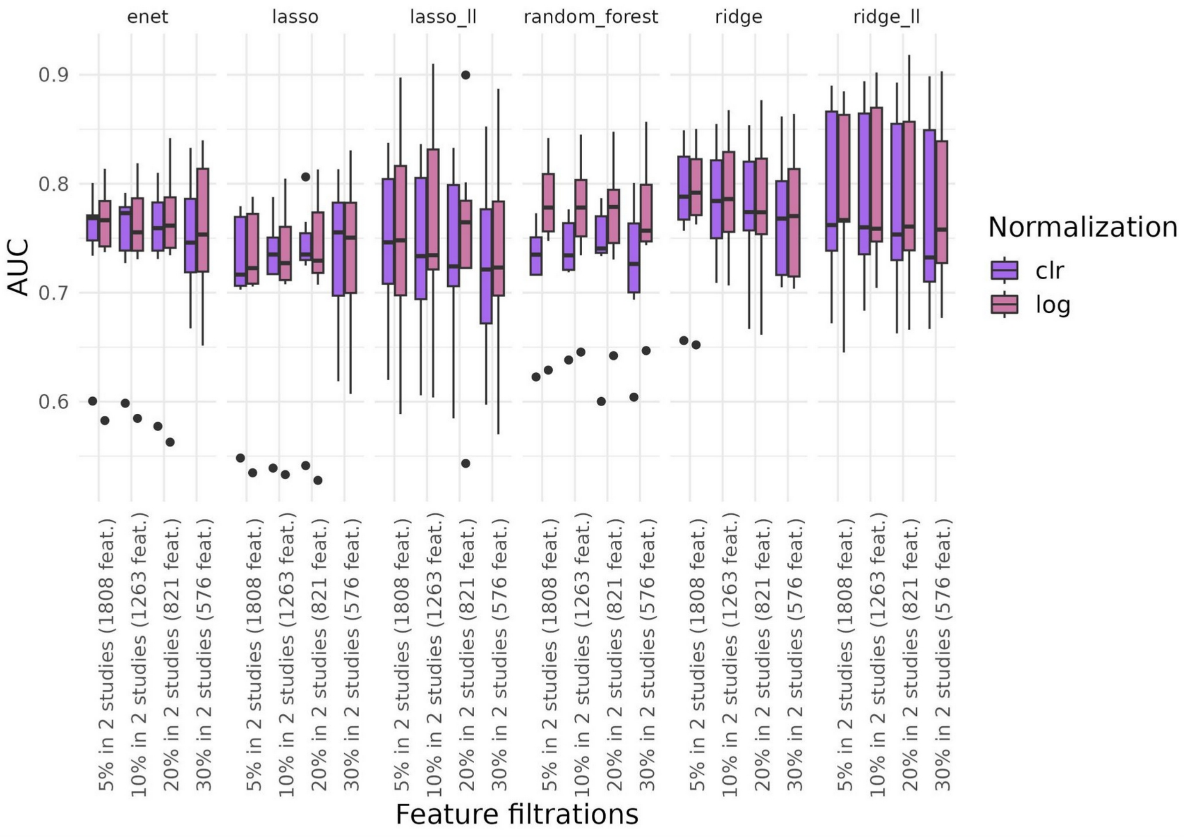

Supplementary Fig. 4 | AUCs for the study-to-study validation (CSV) performed using 16S amplicon data. *Diagonal values indicate the AUCs for the within-study cross-validation (CV). AUCs for the different ML algorithms tested are reported.*

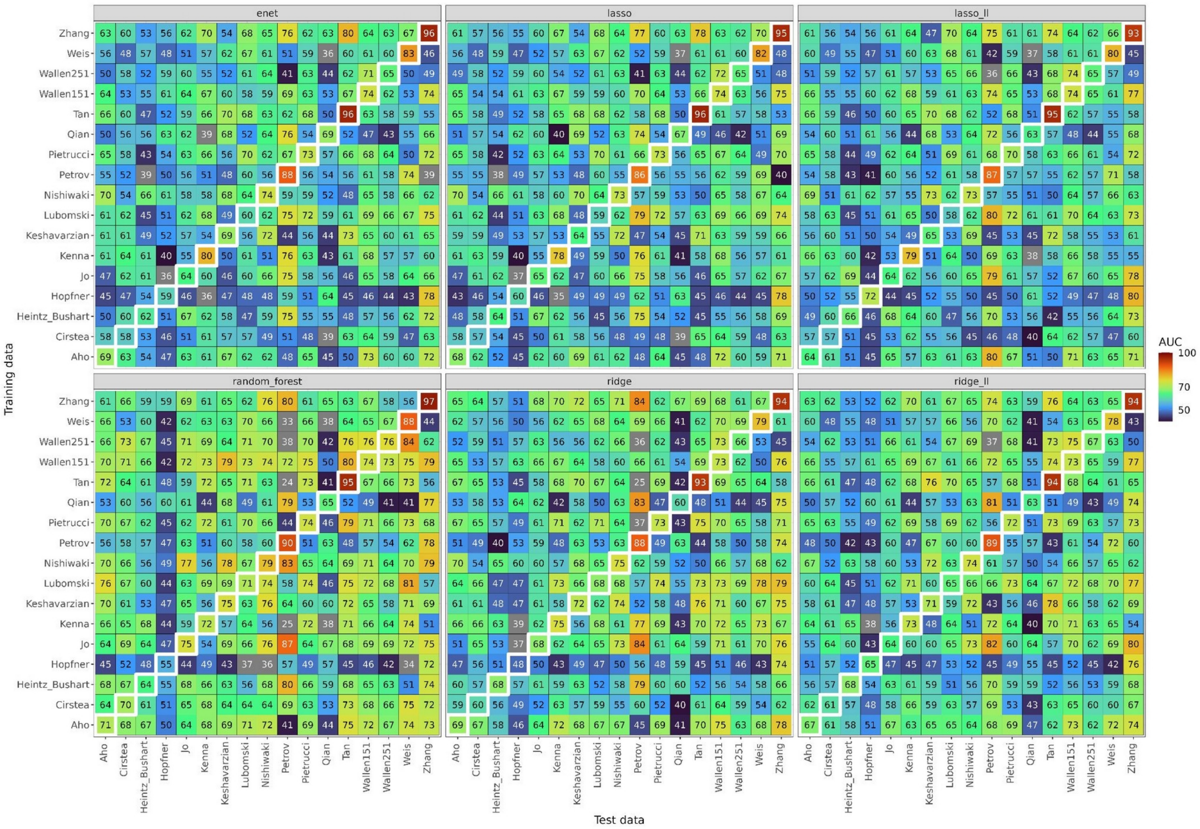

Supplementary Fig. 5 | AUCs for the study-to-study validation (CSV) performed using shotgun metagenomic data. *Diagonal values indicate the AUCs for the within-study cross-validation (CV). AUCs for the different ML algorithms tested are reported.*

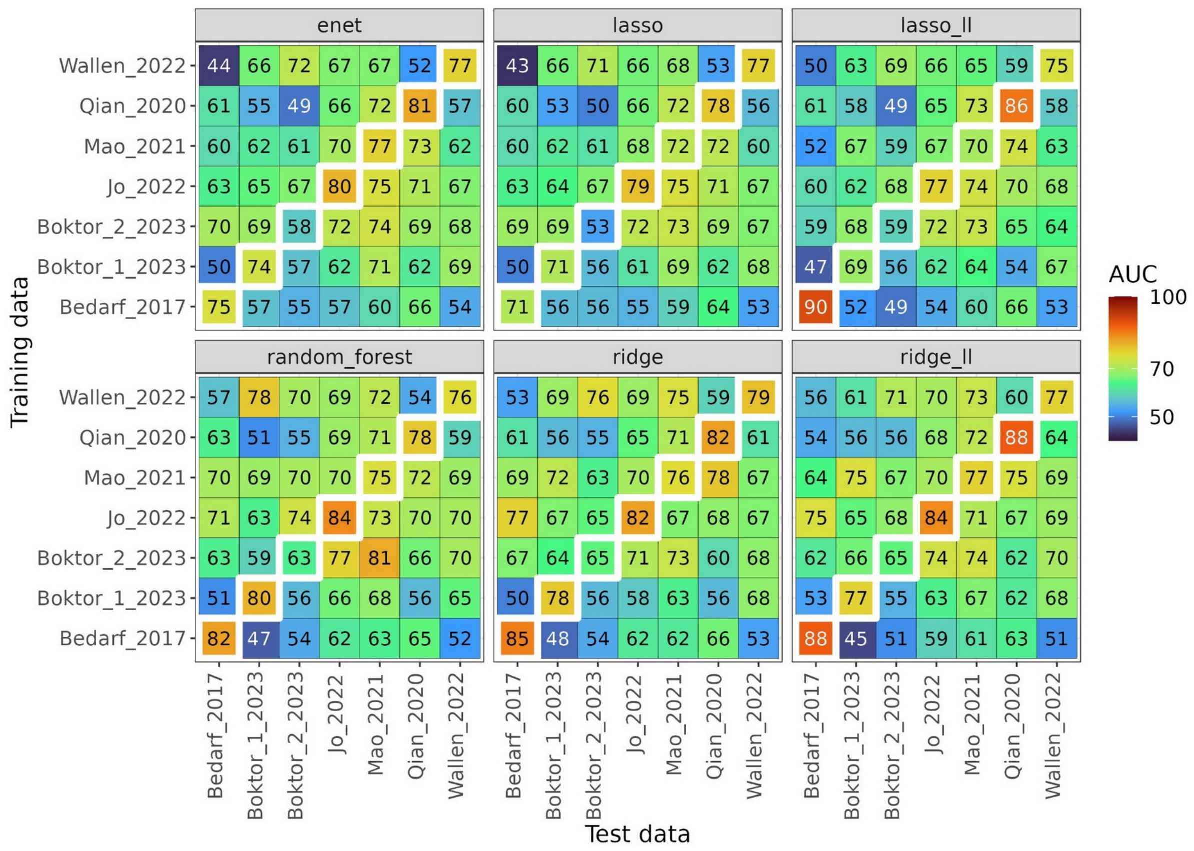

Supplementary Fig. 6 | Prediction accuracies of the taxonomy-based ML models. *Performance of the ML models across datasets for the 16S* ***(a)*** *and SMG* ***(b)*** *data. Blue and yellow symbols indicate the AUCs for the within-study cross-validation (CV) and the leave-one-study-out validation (LOSO), respectively. For the within-study CV data, the AUCs obtained from each of the 10 rounds of 1X10 CV were used to calculate an average AUC (blue dot) and a standard deviation (blue bar). The LOSO validation was instead performed by building 100 models by randomly subsetting 90% of the training set. All these models were then tested on the holdout dataset (test set). The AUCs obtained from each of the 100 models tested on the hold-out dataset were then used to calculate an average AUC (yellow triangle) and a standard deviation (yellow bar). The grey dashed line marks the 50% AUC threshold indicating random guessing.*

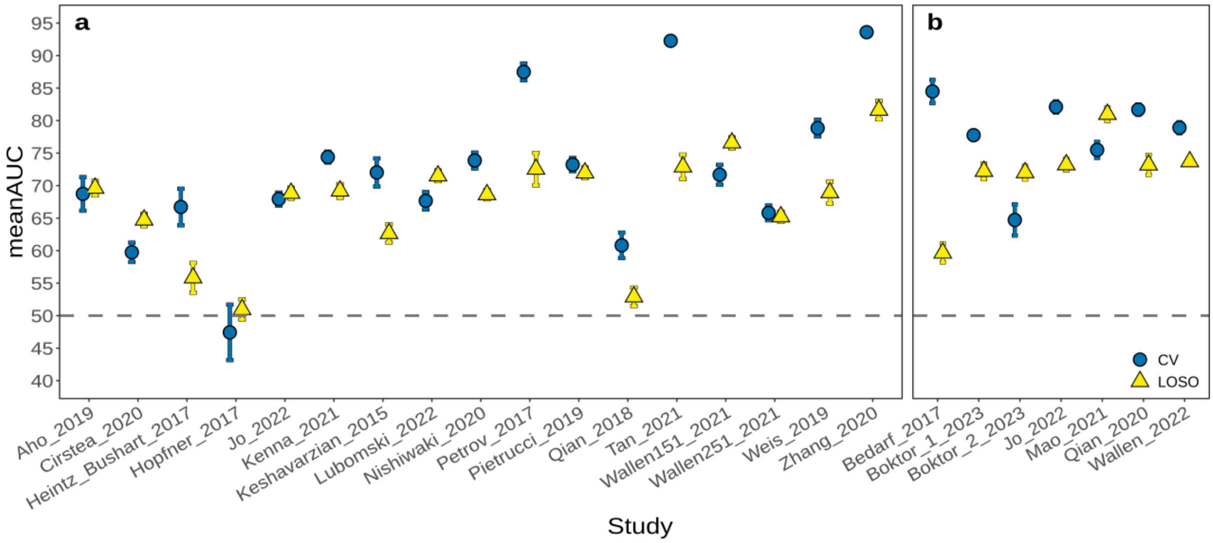

Supplementary Fig. 7 | In comparison across studies, sample size (i.e. training set size) does not significantly influence CV and CSV accuracies. *To contrast the influence of sample size (used as training data) with study effects, we plotted the AUCs against the size of the datasets. This was done for both 16S* ***(a)*** *and SMG* ***(b)*** *data. Segments indicate the CSV AUC ranges, points indicate the median AUC CSV value, and triangles the AUCs for within-study CV. For both 16S and SMG datasets, which have comparable average sample sizes (mean sample size: 16S = 198 ± 189; SMG = 189 ± 238), no cross-study relationship is visible between number of samples and AUCs. We tested for an association between sample size and AUCs across data sets using Pearson correlations, none of which were statistically significant (see main text). For the study of Jo et al.[1] both 16S amplicon and SMG sequencing was performed on a similar number of largely matched individuals (16S = 172 samples; SMG = 156 samples). Here, the SMG data resulted in models with better AUCs in both CV and CSV. A higher CV AUC was maintained even when we matched the samples between 16S and SMG and used them to build models with exactly the same training and test splits (c). Altogether these data indicate that study-specific variation is so large that it masks the theoretically expected gain in AUC with increasing training set size, whereas the differences in accuracies between SMG and 16S are unlikely to be due to the differences in training set size.*

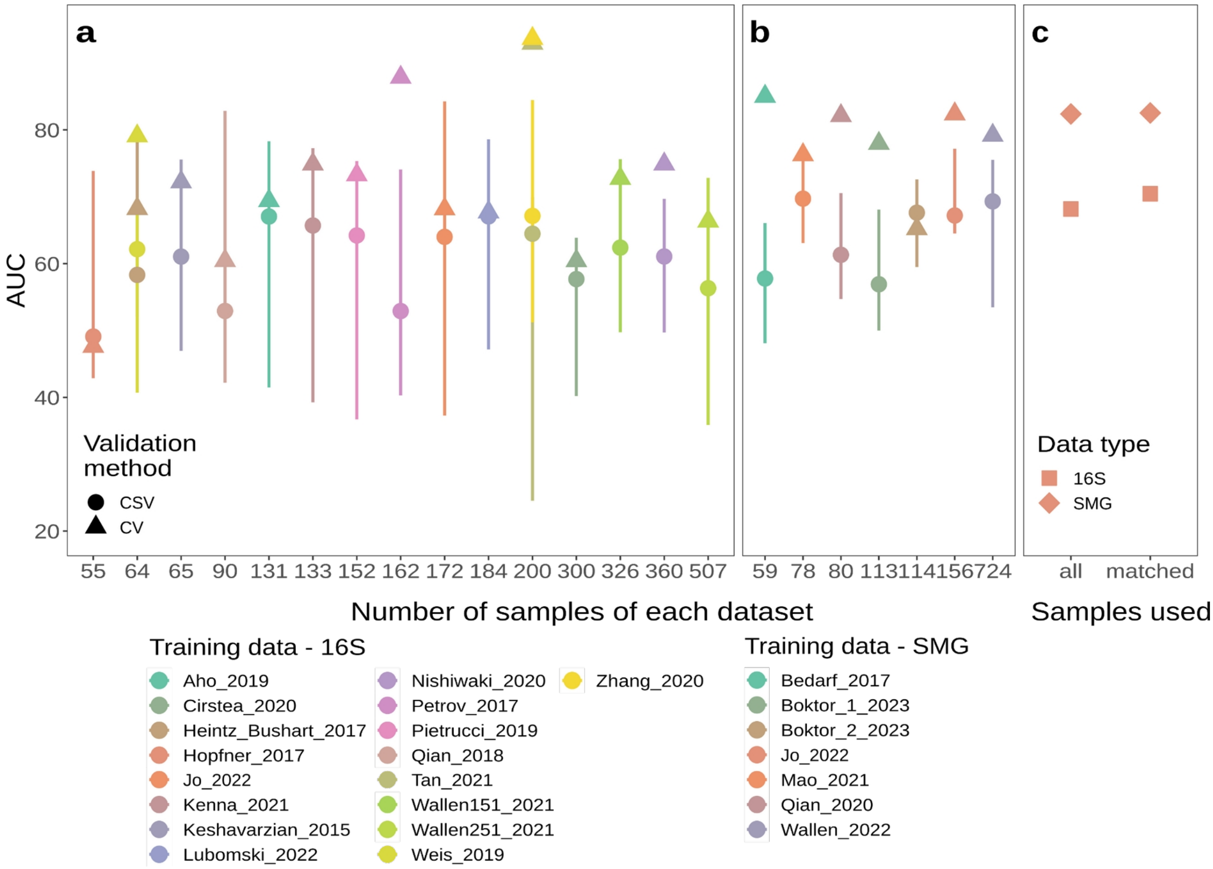

1. Jo, S. *et al.* Oral and gut dysbiosis leads to functional alterations in Parkinson's disease. *Npj Park. Dis.* **8**, 1–12 (2022).

Supplementary Fig. 8 | Influence of sex, age, and geographical origin of the populations on CV and CSV accuracies. *For both 16S **(a-d)** and SMG **(e-h)** taxonomies, we investigated how the study-specific distributions of donor age **(a, d)** and sex **(b, e)** influenced accuracies. This was done for both the within-study CV and the CSVs (in both training and test sets). Age refers to the ratios between the average ages of controls and PD in each dataset. For sex, we first calculated, in each study, female (F) vs. male (M) ratios in both PD and controls. We then used these F/M values to compute new ratios between controls and PD (X-axis). The AUCs obtained from the CSV were then partitioned based on the Western (W) vs Eastern (E) origin of the training and test set **(c, g)**; similarly, we partitioned the within-study CV between the E and W studies **(c, g)**. When training and test data originated from the same geographical area (E_E, W_W) one might expect higher AUCs, but this was not generally observed here. The association between population features and AUCs was tested using linear models (lm) and the proportion of variance explained is reported as R^2 in percent **(d, h)**. None of the associations were statistically significant (16S data: train-set in CSV, gender estimate = 0.8, std. error = 0.9, 95% confidence interval (ci) = -0.9 - 2.5, t = 0.9, p-value = 0.4; age estimate = 6.5, std. error = 8, 95% ci = -9.3 - 22.2, t = 0.8, p-value = 0.4; test-set in CSV, gender estimate = 1.6, std. error = 0.9, 95% ci = -0.3 - 3.4, t = 1.7, p-value = 0.09; age estimate = -0.3, std. error = 8.7, 95% ci = -17.5 - 16.9, t = -0.04, p-value = 0.97; CV, gender estimate = -0.8, std. error = 4.8, 95% ci = -11.8 - 10.1, t = -0.2, p-value = 0.9; age estimate = -25.4, std. error = 43.7, 95% ci = -126.1 - 75.3, t = -0.6, p-value = 0.6. AUCs across geographies, CSV: degrees of freedom = 3, F = 0.9, p-value = 0.5; CV: degrees of freedom = 1, F = 1.8, p-value = 0.2. SMG data: train-set in CSV, gender estimate = 2.5, std. error = 1.5, 95% ci = -0.7 - 5.7, t = 1.6, p-value = 0.1; age estimate = 41.3, std. error = 38.6, 95% ci = -38.6 - 121.3, t = 1.1, p-value = 0.3; test-set in CSV, gender estimate = 0.6, std. error = 1.6, 95% ci = -2.7 - 4, t = 0.4, p-value = 0.7; age estimate = -11.1, std. error = 40, 95% ci = -94 - 71.8, t = -0.3, p-value = 0.8; CV, gender estimate = 1.6, std. error = 2.7, 95% ci = -5.8 - 9, t = 0.6, p-value = 0.6; age estimate = -74.6, std. error = 64.4, 95% ci = -232 - 82.9, t = -1.2, p-value = 0.3. AUCs across geographies, CSV: degrees of freedom = 3, F = 2.4, p-value = 0.08; CV: degrees of freedom = 1, F = 0.4, p-value = 0.6)*

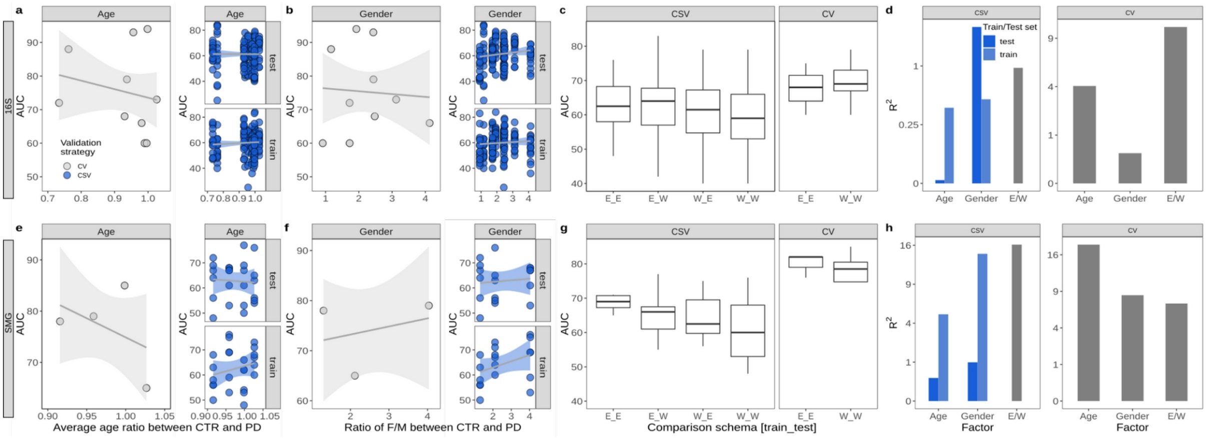

Supplementary Fig. 9 | Number of reads between conditions and across 16S amplicon datasets. *Data distributions are depicted using boxplots, in which 50% of the data (25th to 75th percentile) are within the limits of the box and the thick vertical line within it indicates the data's median (50th percentile). For each dataset n = number of samples reported in Table 1.*

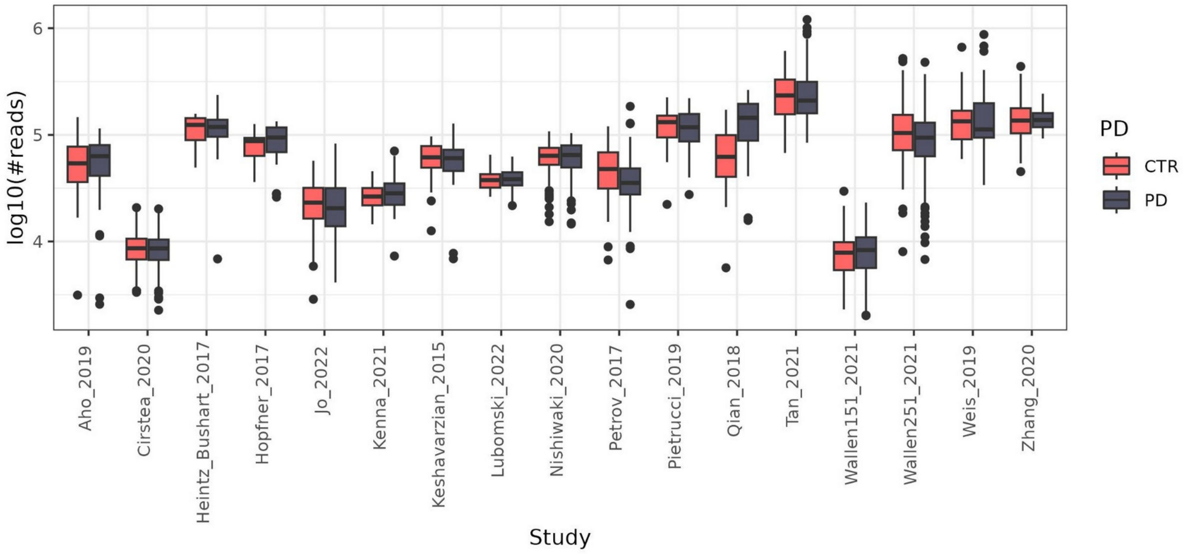

Supplementary Fig. 10 | Performance comparisons of ML models built on rarefied and not rarefied 16S amplicon data. *Data were rarefied to a max depth of 2000, and new ML models were built and used to perform study-to-study validation (CSV). AUC values obtained from the models built for the rarefied and not rarefied data were then compared (n = 272 AUCs). Paired t-tests were performed between the AUCs obtained from the two data types: ENET, t = -1.6, df = 271, p-value = 0.1, effect size = -0.1, 95% confidence interval (ci) = -0.23 - 0.03; LASSO, t = -1.7, df = 271, p-value = 0.1, effect size = -0.1, 95% ci = -0.23 - 0.02; LASSO LibLinear, t = -1.7, df = 271, p-value = 0.1, effect size = -0.1, 95% ci = -0.23 - 0.03; Random Forest, t = 0.05, df = 271, p-value = 0.96, effect size = 0.003, 95% ci = -0.12 - 0.13; Ridge regression, t = -3.2, df = 271, p-value = 0.002, effect size = -0.2, 95% ci = -0.32 - -0.06; Ridge LibLinear, t = -4.1, df = 271, p-value = 0.00005, effect size = -0.25, 95% ci = -0.38 - -0.13.*

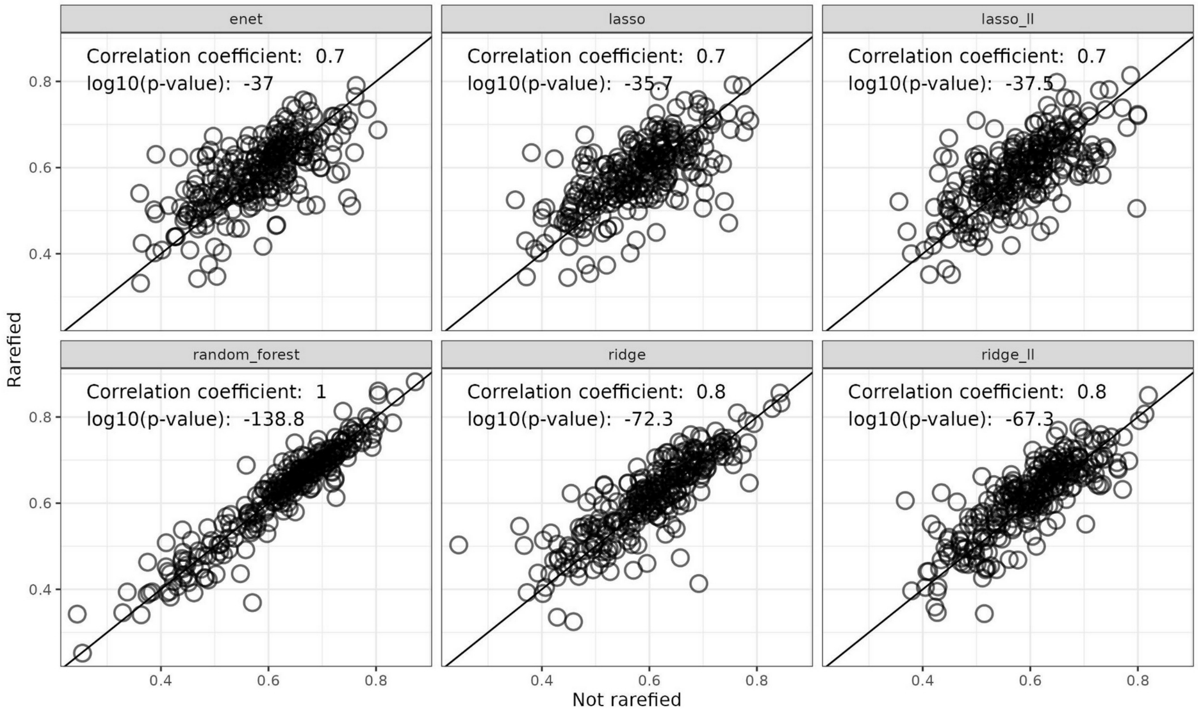

Supplementary Fig. 11 | Performance comparisons of ML models built on raw and batch-corrected 16S amplicon data. *Batch effect was corrected using different label-blind approaches, and the new data were used to build new study-specific ML models which were then used to perform study-to-study validation (CSV). For all algorithms tested, there is a significant difference in AUCs across groups (tested using linear mixed effect models and the training-test set as random intercept: degrees of freedom = 4, ENET F = 26.1, p-value < 0.01; LASSO F = 18.4, p-value = < 0.01; LASSO-LibLinear F = 12.7, p-value = < 0.01; Random Forest F = 35.4, p-value = < 0.01; Ridge regression F = 14, p-value = < 0.01; Ridge-LibLinear F = 16, p-value = < 0.01; n = 272 AUCs). However,* none of the batch correction approaches significantly increased the average AUC in the CSV evaluations (Supplementary data 2). For *details on the different batch correction methods please see the Methods section.*

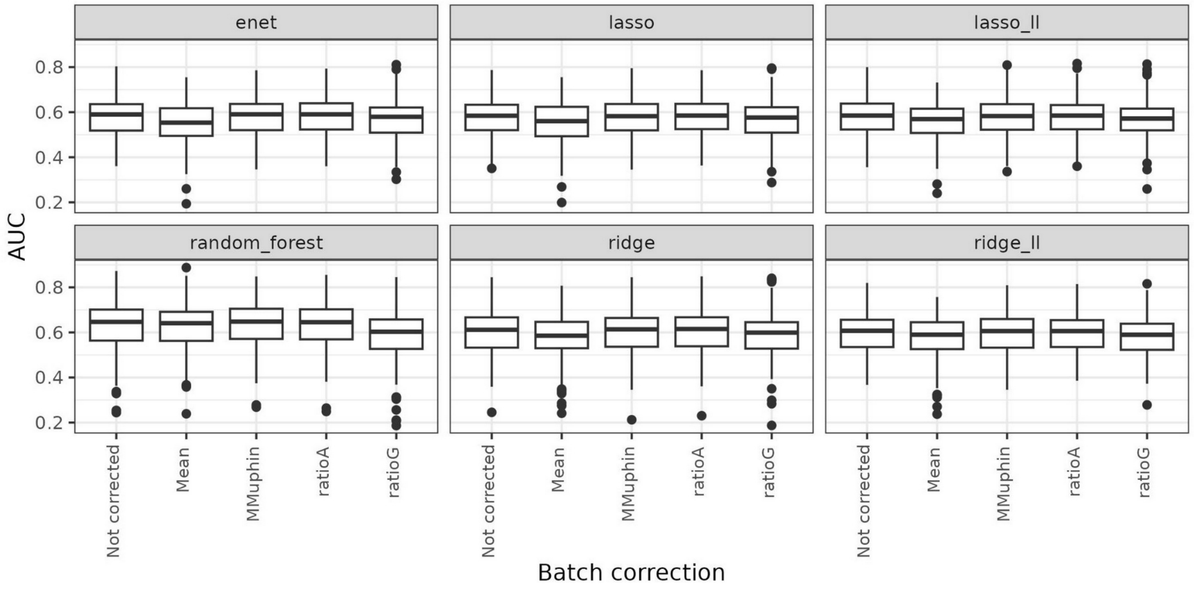

Supplementary Fig. 12 | Increasing the sample size of the training dataset improves AUCs in the LOSO validation. *Seven independent LOSO validations, one for each SMG dataset, were performed using different training sets. One SMG dataset was treated as a holdout and used as a test set (named on the plot strip), and the others were pooled progressively increasing the number of combined sets from 2 to 6 (for all possible combinations). For each test set, we obtained a total of 57 different training sets. The colour and the size of the dots refers to the number of datasets pooled for training. The bar plot shows the proportion of variance explained by the two variables used in a linear model built to test the association between the number of samples and AUCs (estimate = 0.03, std. error = 0.003, 95% confidence interval = 0.02 - 0.03, df = 391, t = 8.7, p-value < 0.001). In the linear model, the number of samples was used as a fixed effect and the test set was used as a random intercept. The proportion of variance explained by the number of samples has been estimated using the marginal R^2 of the linear model. Instead, the proportion of variance explained by the test set was calculated using the intraclass correlation coefficient (see Methods for details).*

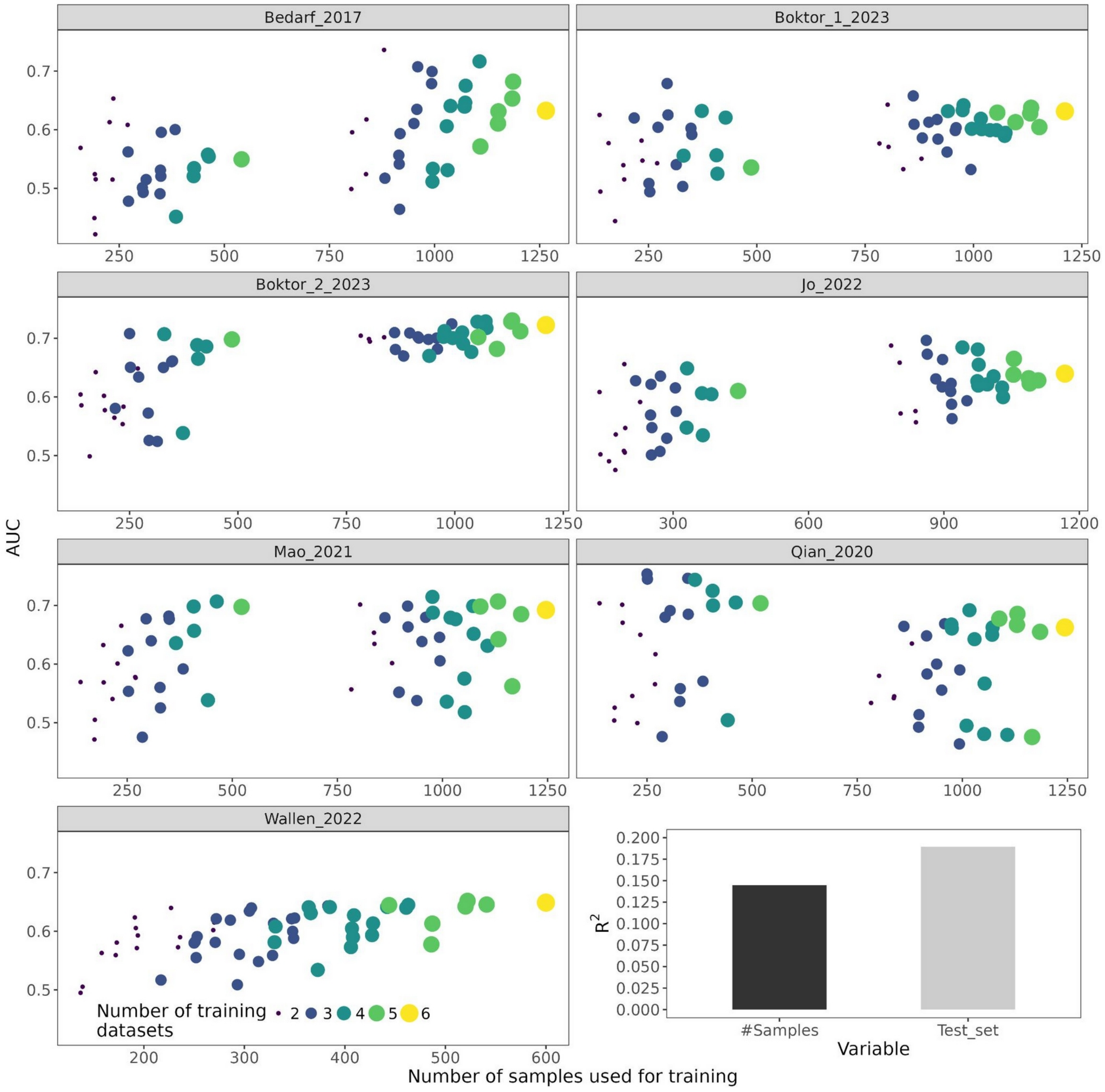

Supplementary Fig. 13 | Ordination based on Ridge regression model weights. *The ordination referring to the 16S amplicon data is reported in panel* ***a*** *and the one referring to shotgun metagenomics data in panel* ***b.*** *Colour of the dots and edges refers to the continent of origin and dataset, respectively. Significance of clustering was assessed using PERMANOVA (16S data: degrees of freedom = 3, R^2 = 18.96%, p-value = 0.4; SMG data: degrees of freedom = 2, R^2 = 34.9, p-value = 0.04).*

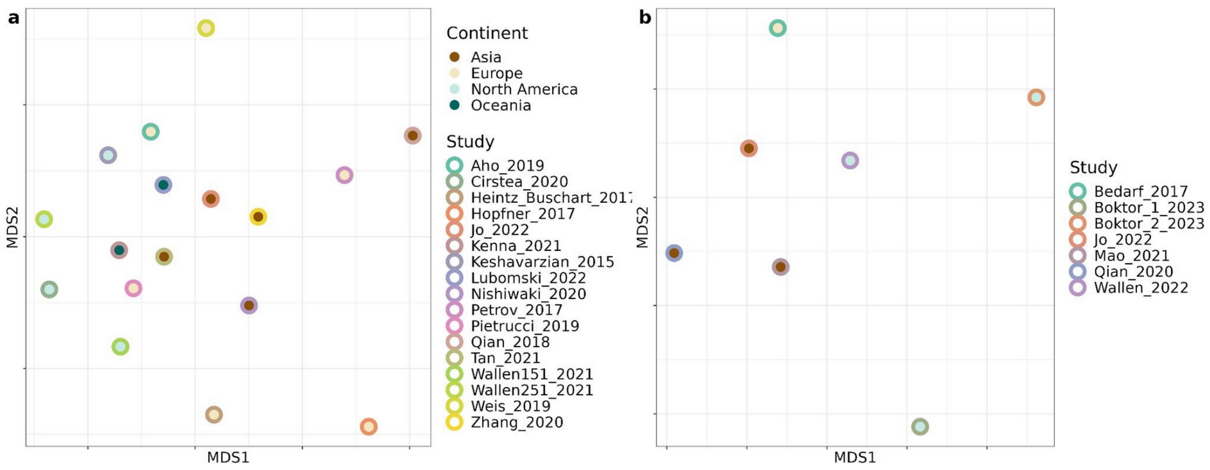

Supplementary Fig. 14 | AUCs for the study-to-study validation (CSV) performed using KEGG orthologous (KO) inferred from the shotgun metagenomics data. *Diagonal values indicate the AUCs for the within-study cross-validation (CV). AUCs for the different ML algorithms tested are reported.*

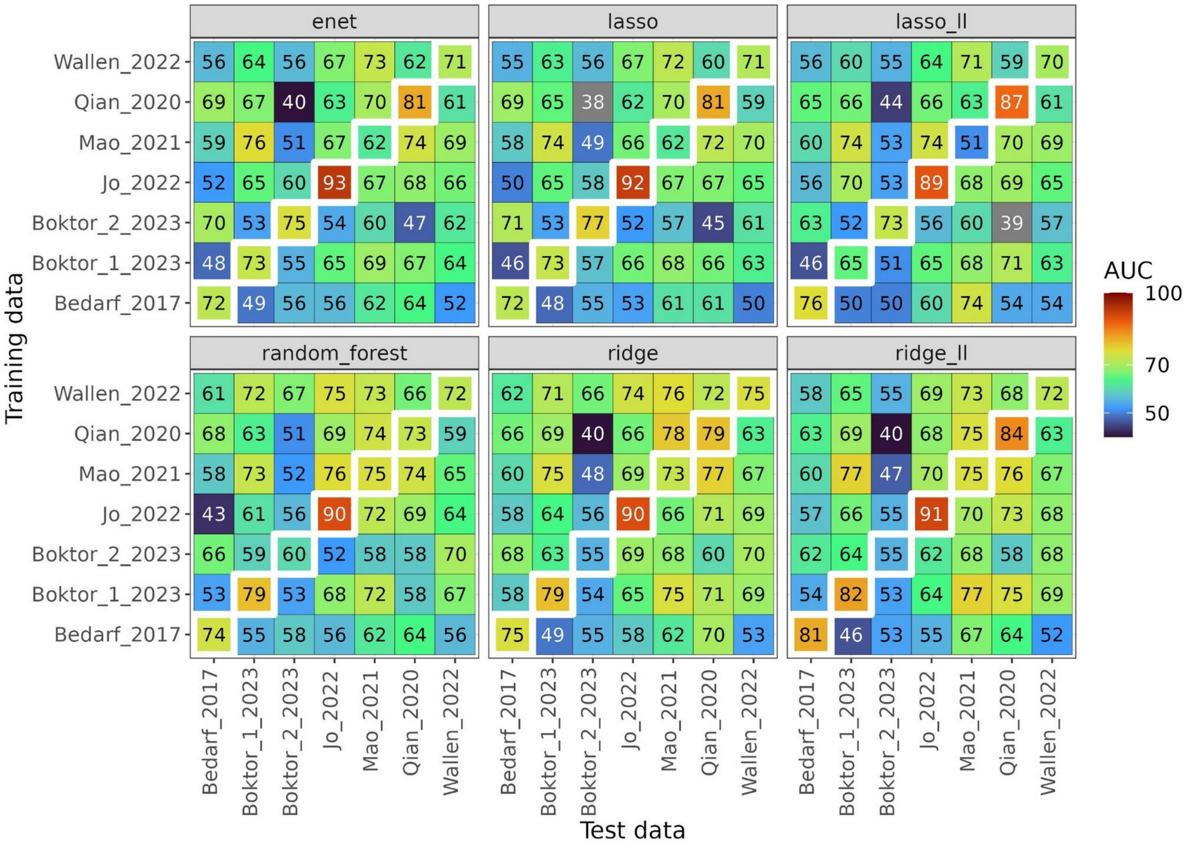

Supplementary Fig. 15 | AUCs for the study-to-study validation (CSV) performed using KEGG modules inferred from the shotgun metagenomics data. *Diagonal values indicate the AUCs for the within-study cross-validation (CV). AUCs for the different ML algorithms tested are reported.*

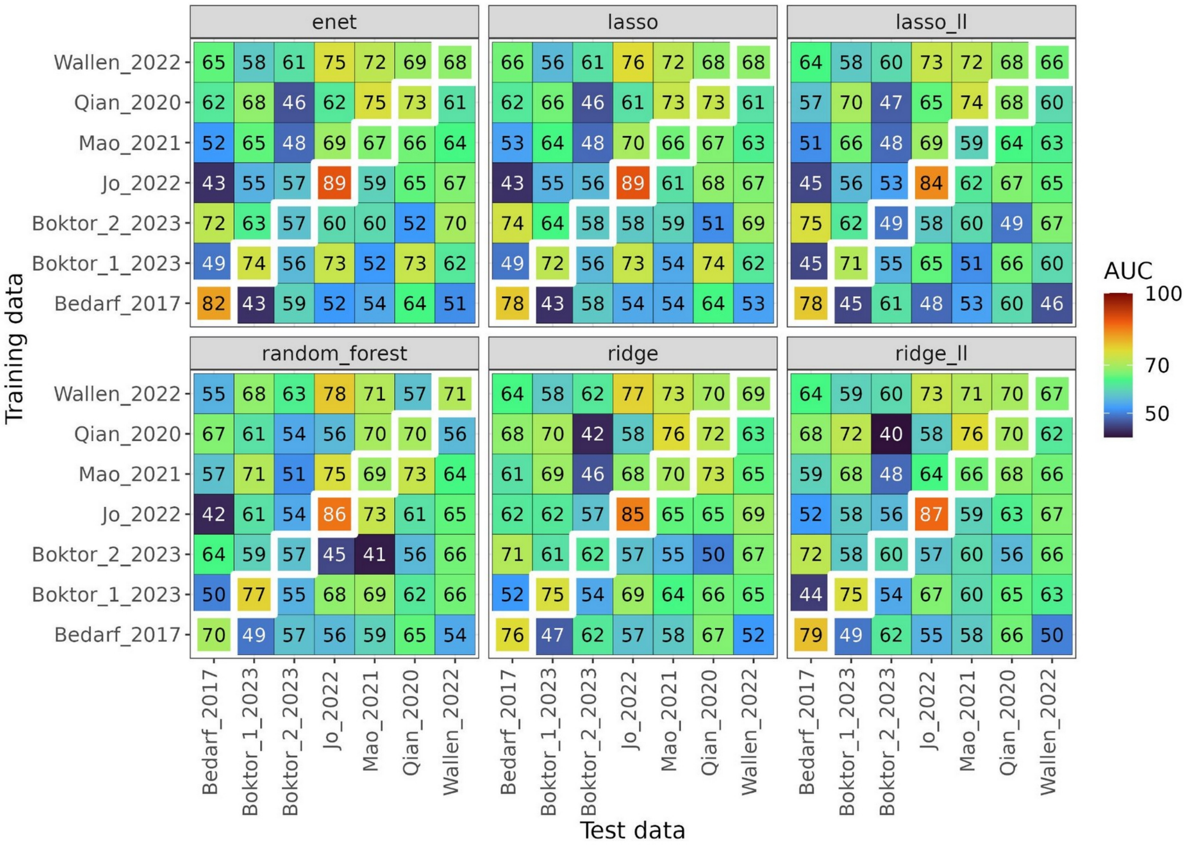

Supplementary Fig. 16 | AUCs for the study-to-study validation (CSV) performed using KEGG pathways inferred from the shotgun metagenomics data. *Diagonal values indicate the AUCs for the within-study cross-validation (CV). AUCs for the different ML algorithms tested are reported.*

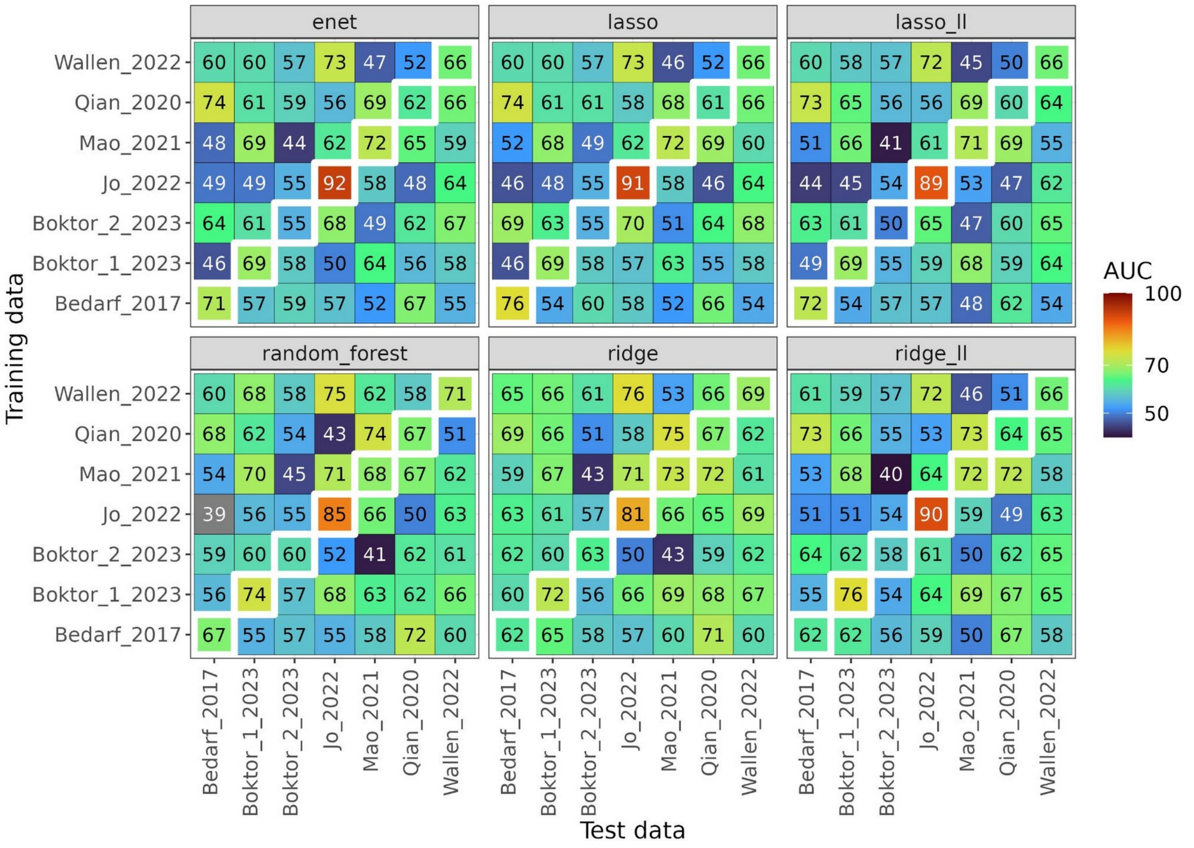

Supplementary Fig. 17 | AUCs for the study-to-study validation (CSV) performed using gut metabolic modules (GMM) inferred from the shotgun metagenomics data. *Diagonal values indicate the AUCs for the within-study cross-validation (CV). AUCs for the different ML algorithms tested are reported.*

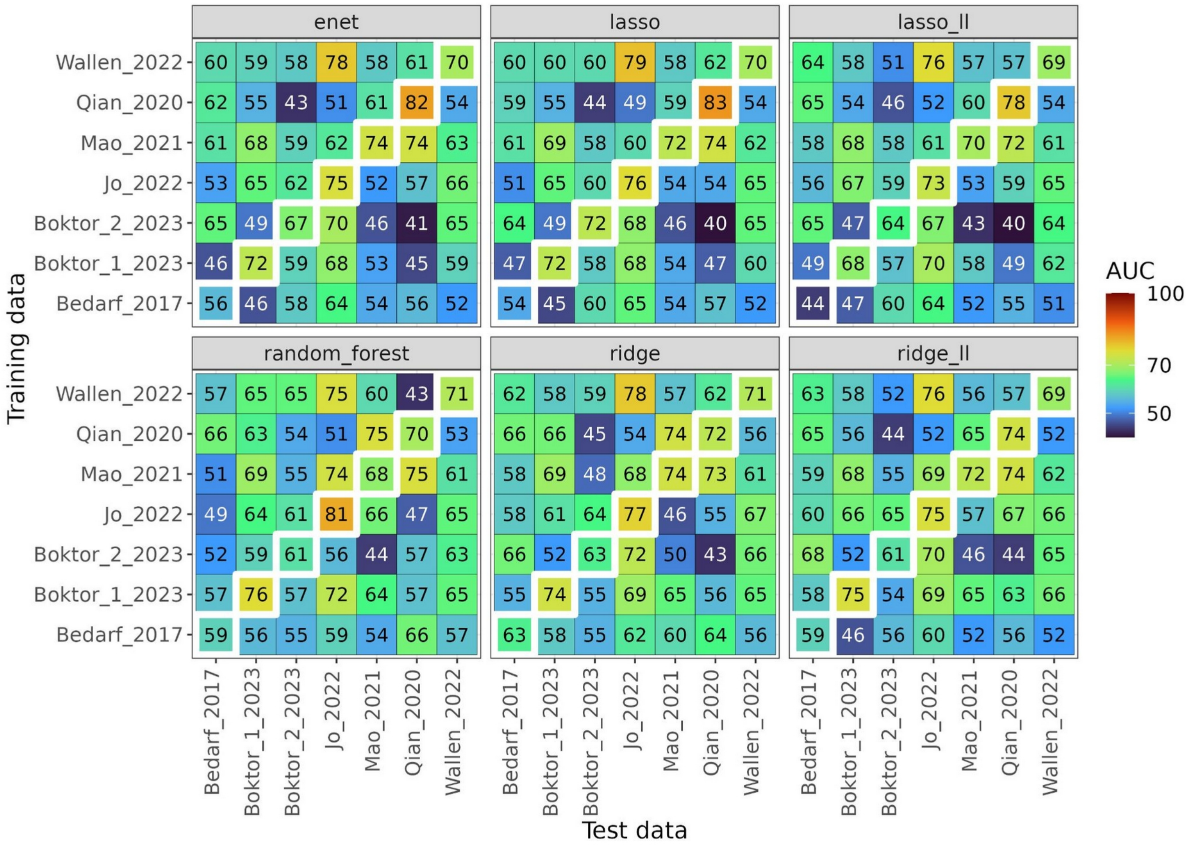

Supplementary Fig. 18 | AUCs for the study-to-study validation (CSV) performed using gut-brain metabolic modules (GBM) inferred from the shotgun metagenomics data. *Diagonal values indicate the AUCs for the within-study cross-validation (CV). AUCs for the different ML algorithms tested are reported*.

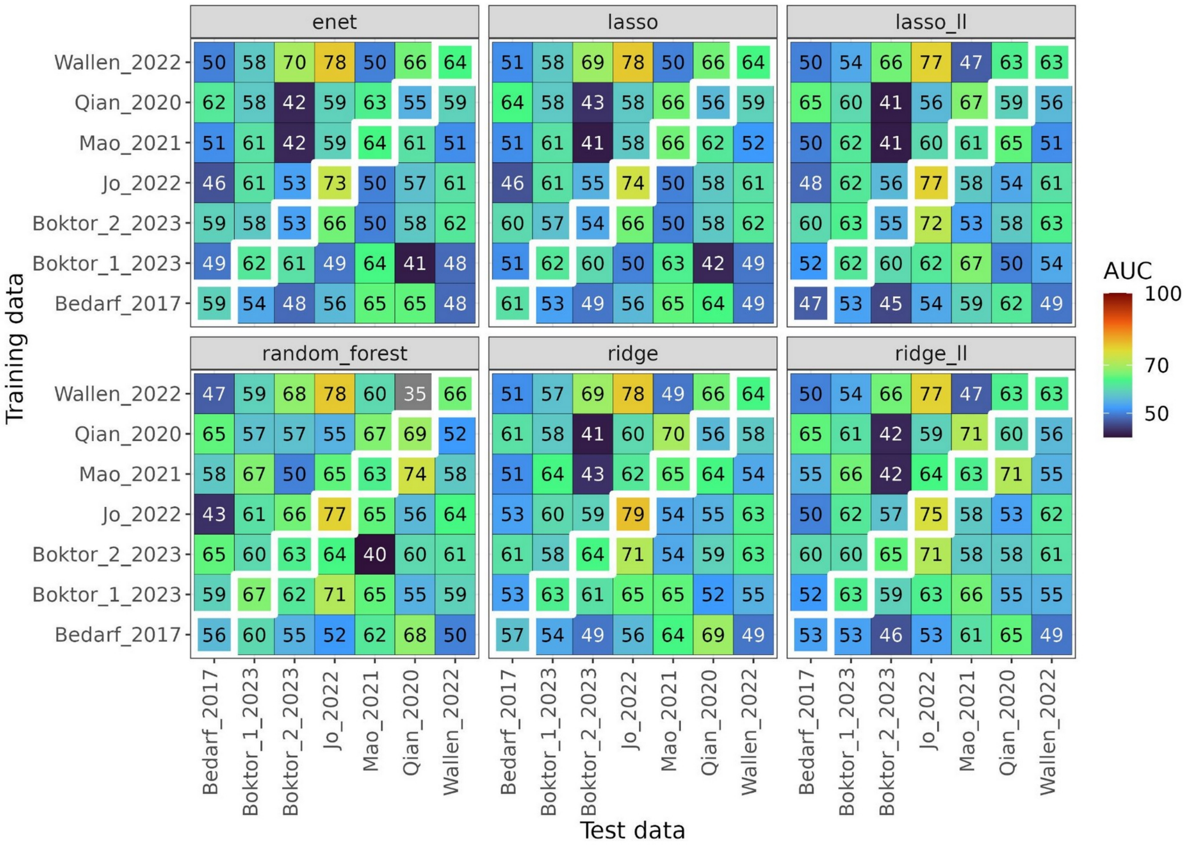

Supplementary Fig. 19 | Ridge regression coefficients strongly correlate with the differential abundance effect sizes. *Average relative Ridge* regression *coefficients computed across all Ridge regression models and Generalised Odds Ratios computed during the differential abundance analyses were plotted against each other and used to estimate Pearson correlations (mOTUs n = 1808; 16S genera n = 212; KEGG KO n = 7605; KEGG pathways n = 144; KEGG modules n = 581; GMM n = 103; GBM n = 49).*

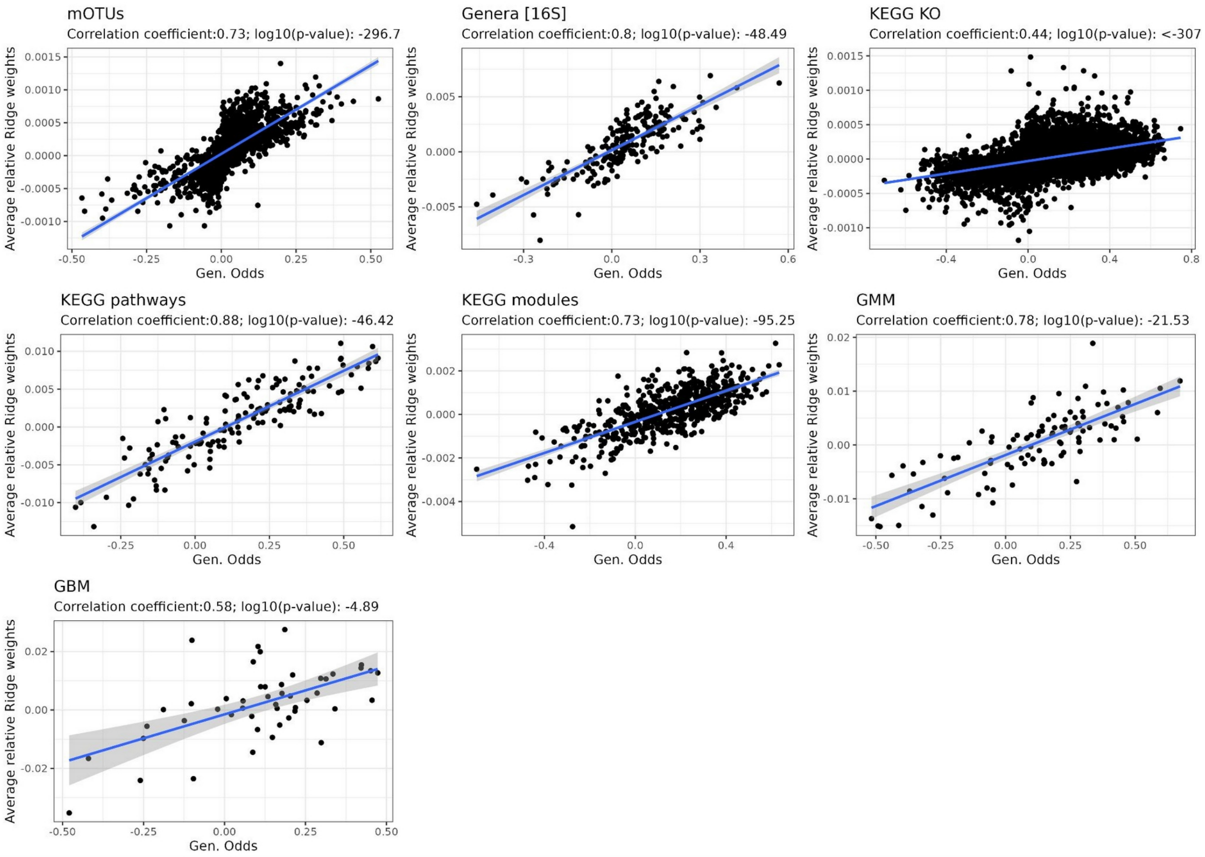

Supplementary Fig. 20 | Proportion of bacterial pathways potentially confounded by covariates. *Heatmap showing the proportion of features associated with PD potentially confounded by sex and age* ***(a)****, general medications* ***(b)****, and PD medications* ***(c)****. KEGG pathways, KEGG modules, and KO are related to the functionalities reported in Fig 7. Grey tiles indicate the absence of specific KEGG modules, within a given pathway, associated with PD. All microbiome features, including both taxa and functions, potentially confounded are reported in Supplementary Data 7-9.*

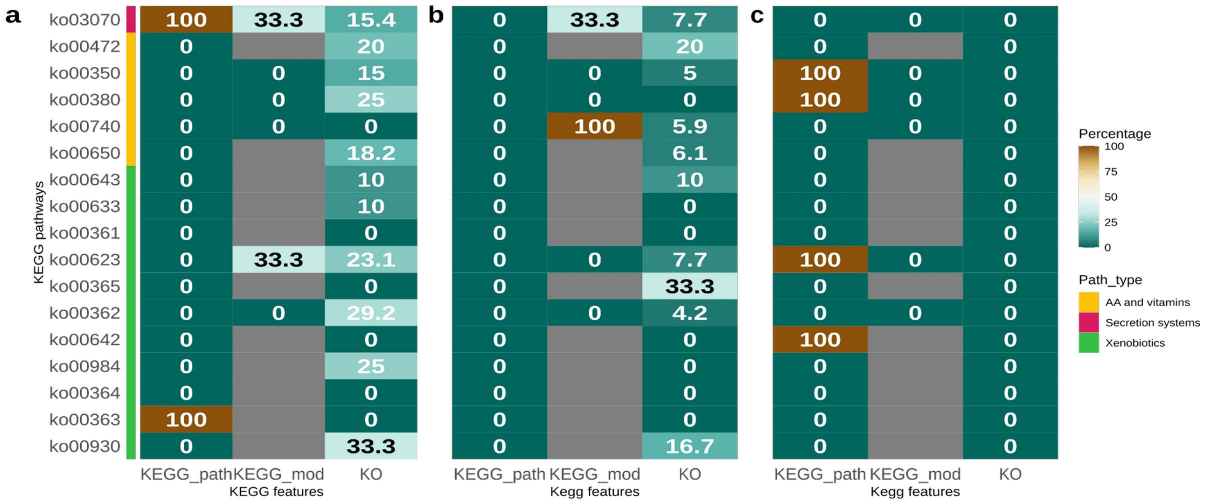

Supplement: Supplementary file 1 — Supplementary Information [file 41467_2025_56829_MOESM1_ESM.pdf]
