# Supplementary material for: Machine learning-based meta-analysis reveals gut microbiome alterations associated with Parkinson’s disease
Source: Nat Commun. 2025 May 7;16:4227. doi: 10.1038/s41467-025-56829-3 (PMC12059030; doi:10.1038/s41467-025-56829-3)
Supplement: Supplementary file 2 — Description of Additional Supplementary Files [file 41467_2025_56829_MOESM2_ESM.pdf]

Description of Additional Supplementary Files

Supplementary Data 1

Permutational multivariate analysis of variance performed on Bray-Curtis dissimilarities. These analyses were performed for both 16S and SMG data. For each analysis, the degrees of freedom (DF), Sum of Squares (Sum of Seqs), F statistic, R squared (R2), and p-values are reported. To test the effect of disease we restricted the permutations within studies.

Supplementary Data 2

Differences in performance between models built on batch-corrected and uncorrected 16S data. For all ML methods we used, AUCs obtained from the CSV performed for each study-specific model were computed. In the table are reported the comparisons (contrasts); the differences in average AUC (estimate) and its respective Standard Errors (SE); lower and upper bounds of the 95% confidence interval (lower.CL and upper.CL; confidence levels were adjusted using the Bonferroni method); degrees of freedom (df); t-statistic (t.ratio); adjusted p-value (adjusted using fdr).

Supplementary Data 3

Differences in performance between models built on different SMG profiles. AUCs obtained for both taxonomic and functional profiles were used to build linear models using the train-test combination as a random intercept. For each validation approach (Validation.type; within-study CV; CSV; LOSO) we compared the AUC distribution across profiles. In the table are reported the comparisons (Contrasts); the differences in average AUC (Estimate) and its respective Standard Errors (SE); degrees of freedom (DF); lower and upper bounds of the 95% confidence interval (lower.CL and upper.CL; confidence levels were adjusted using the Bonferroni method); t-statistic (t.ratio); adjusted p-value (adjusted using fdr).

Supplementary Data 4

Genera showing a significant difference in abundance between PD patients and controls (CTRL). Results of the differential abundance analysis performed independently for each dataset using Agresti Generalised Odd ratios on the genera obtained from the 16S amplicon data. Results were pooled using random effect meta-analysis. The number of datasets in which each genus was significantly enriched in PD and in CTRL is reported in the columns times.sign.PD and times.sign.CTRL, respectively. In addition, for each genus we report the average ridge weight and its standard deviation (average.ridge.weight and stdev.ridge.weight), calculated considering the weights obtained for the ridge models built on each dataset. mOTUs = correspondent mOTUs detected differentially abundant between conditions in the shot-gun metagenomic data; effect.size = generalised odd ratios pooled using random effect meta-analysis; SE = standard deviation of the effect size; CI.upper = upper limit of the effect size 95% confidence interval; CI.lower = lower limit of the effect size 95% confidence interval;

direction.enrich = description on whether the taxon was enriched in PD or CTRL; p.value.meta = p-values obtained from the random effect meta-analysis; q.value = fdr corrected p-values.

Supplementary Data 5

mOTUs showing a significant difference in abundance between PD patients and controls (CTRL). Results of the differential abundance analysis performed independently for each dataset using Agresti Generalised Odd ratios on the mOTUs obtained from the shot-gun metagenomic data. Results were pooled using random effect meta-analysis. The number of datasets in which each mOTU was significantly enriched in PD and in CTRL is reported in the columns times.sign.PD and times.sign.CTRL, respectively. In addition, for each mOTU we report the average ridge weight and its standard deviation (average.ridge.weight and stdev.ridge.weight), calculated considering the weights obtained for the ridge models built on each dataset. Genera_16S = correspondent genus inferred using the 16S amplicon data, detected differentially abundant between conditions; effect.size = generalised odd ratios pooled using random effect meta-analysis; SE = standard deviation of the effect size; CI.upper = upper limit of the effect size 95% confidence interval; CI.lower = lower limit of the effect size 95% confidence interval; direction.enrich = description on whether the taxon was enriched in PD or CTRL; p.value.meta = p-values obtained from the random effect meta-analysis; q.value = fdr corrected p-values.

Supplementary Data 6

Percentages of microbiome features associated with PD potentially confounded by sex, age, or medication usage. The microbiome features we detected associated with PD (either enriched or depleted) in the meta-analysis were used to perform a sensitivity analysis to assess the effect of covariates on the associations. Proportion of potentially confounded microbiome features = prop.conf. Type of confounders (sex, age, or medications) = pot.conf. Data type = seqs.

Supplementary Data 7

Microbiome features associated with PD potentially confounded by medication usage. All microbiome features enriched or depleted in PD in our meta-analysis have been used to perform a sensitivity analysis. All these features were used to verify whether their association with PD might be potentially confounded by medication usage. For each feature (Microbiome.feature) we checked the association with PD before and after accounting for confounding medications. Linear models with and without covariates were run using the data from Wallen et al.1 and p-values were corrected using false discovery rate (fdr). p.value.cov.PD = p-value for the PD estimate in the linear model accounting for covariates; p.value.cov.PD.adj = p-values adjusted using fdr (q-values); est.cov.PD = beta coefficient for PD in the linear model built accounting for covariates; p.value.PD = p-value for the PD estimate in the linear model not including covariates; p.value.PD.adj = p-values adjusted using fdr (q-values); est.PD = beta coefficient for PD in the linear model built not accounting for covariates. The description and the type of microbiome features are reported in the columns description and type, respectively. Finally, we report the variables that were used to build the final models after variable selection based on Mallows's Cp.

Supplementary Data 8

Microbiome features showing associations with PD medications. We tested the association of all microbiome features enriched or depleted in PD in our meta-analysis with PD medication usage. For each medication p-values and estimates (as calculated using linear models) are reported (".pvale"; ".est"). We selected all significant associations with any medication and report in Est.drug.concordance whether the drugs had similar or opposite effects on feature abundances (Neg = features abundance decreases with drug usage; Pos = feature abundance increases with drug usage; Disc = discordant effect size between drugs). Similarly, we report in Est.drug.PD.concordance whether PD drugs had a similar or opposite effect than PD on feature abundances. Features types and descriptions of features for the functional ones are reported in the columns description and feature.type, respectively.

Supplementary Data 9

Microbiome features associated with PD potentially confounded by sex and age. All microbiome features enriched or depleted in PD in our meta-analysis have been used to verify whether their association with PD might be potentially confounded by sex and age. For each feature (Microbiome.feature) we checked the association with PD before and after accounting for confounders. P-values for both the linear models with and without covariates were corrected using false discovery rate (fdr). p.value.cov.PD = p-value for the PD estimate in the linear model accounting for covariates; p.value.cov.PD.adj = p-values adjusted using fdr (q-values); est.cov.PD = beta coefficient for PD in the linear model built accounting for covariates; p.value.PD = p-value for the PD estimate in the linear model not including covariates; p.value.PD.adj = p-values adjusted using fdr (q-values); est.PD = beta coefficient for PD in the linear model built not accounting for covariates. We report also all p-values (p.values.sex, p.value.age), q-values (p.values.sex.adj, p.value.age.adj), and estimates (est.sex, est.age) for the sex and age variables. The description and the type of microbiome features are reported in the columns description and type, respectively.

Supplementary Data 10

KEGG orthologous (KOs) showing a significant difference in abundance between PD patients and controls (CTRL). Results of the differential abundance analysis performed independently for each dataset using Agresti Generalised Odd ratios on the KOs obtained from the shot-gun metagenomic data. Results were pooled using random effect meta-analysis. The number of datasets in which each KO was significantly enriched in PD and in CTRL is reported in the columns times.sign.PD and times.sign.CTRL, respectively. In addition, for each KO we report the average ridge weight and its standard deviation (average.ridge.weight and stdev.ridge.weight), calculated considering the weights obtained for the ridge models built on each dataset. effect.size = generalised odd ratios pooled using random effect meta-analysis; SE = standard deviation of the effect size; CI.upper = upper limit of the effect size 95% confidence interval; CI.lower = lower limit of the effect size 95% confidence interval; direction.enrich = description

on whether the KO was enriched in PD or CTRL; p.value.meta = p-values obtained from the random effect meta-analysis; q.value = fdr corrected p-values.

Supplementary Data 11

KEGG modules showing a significant difference in abundance between PD patients and controls (CTRL). Results of the differential abundance analysis performed independently for each dataset using Agresti Generalised Odd ratios on the modules obtained from the shot-gun metagenomic data. Results were pooled using random effect meta-analysis. The number of datasets in which each module was significantly enriched in PD and in CTRL is reported in the columns times.sign.PD and times.sign.CTRL, respectively. In addition, for each module we report the average ridge weight and its standard deviation (average.ridge.weight and stdev.ridge.weight), calculated considering the weights obtained for the ridge models built on each dataset. effect.size = generalised odd ratios pooled using random effect meta-analysis; SE = standard deviation of the effect size; CI.upper = upper limit of the effect size 95% confidence interval; CI.lower = lower limit of the effect size 95% confidence interval; direction.enrich = description on whether the module was enriched in PD or CTRL; p.value.meta = p-values obtained from the random effect meta-analysis; q.value = fdr corrected p-values.

Supplementary Data 12

KEGG pathways showing a significant difference in abundance between PD patients and controls (CTRL). Results of the differential abundance analysis performed independently for each dataset using Agresti Generalised Odd ratios on the pathways obtained from the shot-gun metagenomic data. Results were pooled using random effect meta-analysis. The number of datasets in which each pathway was significantly enriched in PD and in CTRL is reported in the columns times.sign.PD and times.sign.CTRL, respectively. In addition, for each pathway we report the average ridge weight and its standard deviation (average.ridge.weight and stdev.ridge.weight), calculated considering the weights obtained for the ridge models built on each dataset. effect.size = generalised odd ratios pooled using random effect meta-analysis; SE = standard deviation of the effect size; CI.upper = upper limit of the effect size 95% confidence interval; CI.lower = lower limit of the effect size 95% confidence interval; direction.enrich = description on whether the pathway was enriched in PD or CTRL; p.value.meta = p-values obtained from the random effect meta-analysis; q.value = fdr corrected p-values.

Supplementary Data 13

Gut microbial modules (GMM) pathways showing a significant difference in abundance between PD patients and controls (CTRL). Results of the differential abundance analysis performed independently for each dataset using Agresti Generalised Odd ratios on the GMM obtained from the shot-gun metagenomic data. Results were pooled using random effect meta-analysis. The number of datasets in which each GMM was significantly enriched in PD and in CTRL is reported in the columns times.sign.PD and times.sign.CTRL, respectively. In addition, for each module we report the average ridge weight and its standard deviation (average.ridge.weight and stdev.ridge.weight), calculated considering the weights obtained for the ridge models built on each dataset. effect.size = generalised odd ratios pooled using

random effect meta-analysis; SE = standard deviation of the effect size; CI.upper = upper limit of the effect size 95% confidence interval; CI.lower = lower limit of the effect size 95% confidence interval; direction.enrich = description on whether the GMM was enriched in PD or CTRL; p.value.meta = p-values obtained from the random effect meta-analysis; q.value = fdr corrected p-values.

Supplementary Data 14

Gut-brain axis modules (GBM) pathways showing a significant difference in abundance between PD patients and controls (CTRL). Results of the differential abundance analysis performed independently for each dataset using Agresti Generalised Odd ratios on the GBM obtained from the shot-gun metagenomic data. Results were pooled using random effect meta-analysis. The number of datasets in which each GBM was significantly enriched in PD and in CTRL is reported in the columns times.sign.PD and times.sign.CTRL, respectively. In addition, for each module we report the average ridge weight and its standard deviation (average.ridge.weight and stdev.ridge.weight), calculated considering the weights obtained for the ridge models built on each dataset. effect.size = generalised odd ratios pooled using random effect meta-analysis; SE = standard deviation of the effect size; CI.upper = upper limit of the effect size 95% confidence interval; CI.lower = lower limit of the effect size 95% confidence interval; direction.enrich = description on whether the GBM was enriched in PD or CTRL; p.value.meta = p-values obtained from the random effect meta-analysis; q.value = fdr corrected p-values.

Supplementary Data 15

Enrichment analysis performed for the KEGG pathways. All KOs enriched in either PD or CTRL were separately used to perform an enrichment analysis. KEGG_ID = KEGG pathways ID; GeneRatio = ratio of KOs within the pathway and all enriched KOs in PD or CTRL; BgRatio = ratios between KOs within the pathway and all KOs considered; p-values = p-values of the hypergeometric test; p.adjust = fdr corrected p-values; geneID = KOs enriched within the pathway; count = count of genes within the pathway; description = name of the KEGG pathway; enrichment dir. = condition (PD or CTRL) in which the KOs are enriched.

Supplementary Data 16

Results of the blastp search performed to verify whether the lactic acid-producing bacteria enriched in PD harbor a homologous of the TyrDC encoding gene. Target enzyme = protein matched by the query sequence in the target organism; target taxa = taxon selected as target for the blastp search; max score = the highest alignment score calculated from the sum of the rewards for matched nucleotides and penalties for mismatches and gaps; total score = the sum of alignment scores of all segments from the same subject sequence; query coverage: the percentage of the query sequence that overlaps the target sequence; E value: number of

expected hits of similar quality (score) that could be found just by chance; per. ident: percentage of base pairs that are the same between the sequence of query and that of the target; acc. len.: the number of amino acids in the target sequence; accession: unique identifier assigned to the target sequence in the NCBI databases; datasets: indicate whether the target taxa has been detected enriched in the 16S or SMG data. For the genera enriched in the 16S data, we report here only the first best hit of the blastp search.
