# Supplementary material for: Machine learning-based meta-analysis reveals gut microbiome alterations associated with Parkinson’s disease
Source: Nat Commun. 2025 May 7;16:4227. doi: 10.1038/s41467-025-56829-3 (PMC12059030; doi:10.1038/s41467-025-56829-3)
Supplement: Supplementary file 4 — Reporting Summary [file 41467_2025_56829_MOESM4_ESM.pdf]

nature portfolio

Corresponding author(s): Stefano Romano, Georg Zeller

Last updated by author(s): Jan 6, 2025

Reporting Summary

Nature Portfolio wishes to improve the reproducibility of the work that we publish. This form provides structure for consistency and transparency in reporting. For further information on Nature Portfolio policies, see our Editorial Policies and the Editorial Policy Checklist.

Statistics

For all statistical analyses, confirm that the following items are present in the figure legend, table legend, main text, or Methods section.

n/a	Confirmed	
☐	☒	The exact sample size (n) for each experimental group/condition, given as a discrete number and unit of measurement
☒	☐	A statement on whether measurements were taken from distinct samples or whether the same sample was measured repeatedly
☐	☒	The statistical test(s) used AND whether they are one- or two-sided<br>*Only common tests should be described solely by name; describe more complex techniques in the Methods section.*
☐	☒	A description of all covariates tested
☐	☒	A description of any assumptions or corrections, such as tests of normality and adjustment for multiple comparisons
☐	☒	A full description of the statistical parameters including central tendency (e.g. means) or other basic estimates (e.g. regression coefficient) AND variation (e.g. standard deviation) or associated estimates of uncertainty (e.g. confidence intervals)
☐	☒	For null hypothesis testing, the test statistic (e.g. F, t, r) with confidence intervals, effect sizes, degrees of freedom and P value noted<br>*Give P values as exact values whenever suitable.*
☒	☐	For Bayesian analysis, information on the choice of priors and Markov chain Monte Carlo settings
☒	☐	For hierarchical and complex designs, identification of the appropriate level for tests and full reporting of outcomes
☐	☒	Estimates of effect sizes (e.g. Cohen's d, Pearson's r), indicating how they were calculated

Our web collection on statistics for biologists contains articles on many of the points above.

Software and code

Policy information about availability of computer code

Data collection	All data and metadata were obtained either by public databases or directly by the authors that produced them. We report in Table 1 all the information related to the availability of the data. The gut microbiome taxonomic and functional profiles from Parkinson's disease patients generated in this study are available on Zenodo (doi: 10.5281/zenodo.14261087)
Data analysis	All data analysis was performed in R (R v_4.2) and the custom scripts created for this project are publicly available in GitHub (https://github.com/StfnRomano/PD_ML_meta). This is the overall list of softwares and R packages we used: Dada2, GTDB v_207, mOTUs v_3.0, gffquant v_2.10 , bbduk v_38.93, kraken2 v_2.1.2, BWA-MEM v_0.7.17, samtools v_1.14, blastp current online version, Bioconductor v_3.15. R packages: `omixerRpm v_0.3.3`, `phyloseq v_1.40`, `vegan v_2.6.4`, `genodds v_1.1.2`, `meta v_6.2.1`, `leaps v_3.1`, `SIAMCAT v_2.0`,`SIAMCAT v_2.10`, `stats v_4.2.3`, `rtk v_0.2.6.1`, `MMUPHin v_1.10.3`, `bapred v_1.1`, `nlme v_3.1.162`, `emmeans v_1.8.5`, `performance v_0.11`, `ggplot2 v_3.4.4`, `mlr3 v_0.15`, `pROC v_1.18.0`, `mlr3extralearners v_0.6`, `coin v_1.4.2`, `ggnewscale v_0.4.9`, `cowplot v_1.1.1`, `BiocManager v_1.30.20`, `clusterProfiler v_4.4.4`,`rstatix v_0.7.2`, `ggh4x v_0.2.5`, `RColorBrewer v_1.1.3`, `microbiome v_1.18.0`

For manuscripts utilizing custom algorithms or software that are central to the research but not yet described in published literature, software must be made available to editors and reviewers. We strongly encourage code deposition in a community repository (e.g. GitHub). See the Nature Portfolio guidelines for submitting code & software for further information.

Data

Policy information about availability of data

All manuscripts must include a data availability statement. This statement should provide the following information, where applicable:

- Accession codes, unique identifiers, or web links for publicly available datasets
- A description of any restrictions on data availability
- For clinical datasets or third party data, please ensure that the statement adheres to our policy

All data used in the article are either publicly available or have been directly obtained from the authors of the original publications, as specifiedsummirized in Table 1. PRJNA601994 [https://www.ebi.ac.uk/ena/browser/view/PRJNA601994]; CRA001938 [https://ngdc.cncb.ac.cn/gsa/search?searchTerm=CRA001938]; PRJNA494620 [https://www.ebi.ac.uk/ena/browser/view/PRJNA494620]; PRJNA381395 [https://www.ebi.ac.uk/ena/browser/view/PRJNA381395]; PRJNA391524 [https://www.ebi.ac.uk/ena/browser/view/PRJNA391524]; PRJNA268515 [https://www.ebi.ac.uk/ena/browser/view/PRJNA268515]; PRJNA510730 [https://www.ebi.ac.uk/ena/browser/view/PRJNA510730]; PRJEB27564 [https://www.ebi.ac.uk/ena/browser/view/PRJEB27564]; PRJEB30615 [https://www.ebi.ac.uk/ena/browser/view/PRJEB30615]; PRJEB14928 [https://www.ebi.ac.uk/ena/browser/view/PRJEB14928]; DRA009229 [https://ddbj.nig.ac.jp/search/entry/sra-submission/DRA009229]; PRJNA808166 [https://www.ebi.ac.uk/ena/browser/view/PRJNA808166]; Kenna et al. [https://doi.org/10.6084/m9.figshare.14345513.v1]; PRJNA742875 [https://www.ebi.ac.uk/ena/browser/view/PRJNA742875]; PRJEB17784 [https://www.ebi.ac.uk/ena/browser/view/PRJEB17784]; PRJNA433459 [https://www.ebi.ac.uk/ena/browser/view/PRJNA433459]; PRJNA588035 [https://www.ebi.ac.uk/ena/browser/view/PRJNA588035]; PRJNA743718 [https://www.ebi.ac.uk/ena/browser/view/PRJNA743718]; PRJNA834801 [https://www.ebi.ac.uk/ena/browser/view/PRJNA834801]; ERP138197 [https://www.ebi.ac.uk/ena/browser/view/PRJEB53401]; ERP138199 [https://www.ebi.ac.uk/ena/browser/view/PRJEB53403]; PRJNA489760 [https://www.ebi.ac.uk/ena/browser/view/PRJNA489760]; PRJNA633959 [https://www.ebi.ac.uk/ena/browser/view/PRJNA633959]; PRJNA321051 [https://www.ebi.ac.uk/ena/browser/view/PRJNA321051]; PRJNA450340 [https://www.ebi.ac.uk/ena/browser/view/PRJNA450340]; PRJEB34168 [https://www.ebi.ac.uk/ena/browser/view/PRJEB34168]; PRJNA721421 [https://www.ebi.ac.uk/ena/browser/view/PRJNA721421]; PRJEB99111 [https://www.ebi.ac.uk/ena/browser/view/PRJEB99111]; PRJNA554111 [https://www.ebi.ac.uk/ena/browser/view/PRJNA554111]; PRJNA734525 [https://www.ebi.ac.uk/ena/browser/view/PRJNA734525]; PRJEB51982 [https://www.ebi.ac.uk/ena/browser/view/PRJEB51982]; metadata Boktor et al. [https://zenodo.org/records/7183678]; metadata Wallen et al. [https://zenodo.org/records/7246185]. The gut microbiome taxonomic and functional profiles from Parkinson's disease patients generated in this study are available on Zenodo (doi: 10.5281/zenodo.14261087).

Research involving human participants, their data, or biological material

Policy information about studies with human participants or human data. See also policy information about sex, gender (identity/presentation), and sexual orientation and race, ethnicity and racism.

Reporting on sex and gender	We used the metadata made available by the studies we re-analyzed. For a clear explanation of criteria and procedure for data collection we encourage to consult the original publication that produced the data. We performed a covariate analysis using sex.
Reporting on race, ethnicity, or other socially relevant groupings	We did not consider this information in our work, as the corresponding data is not available for all datasets we re-analyzed. However, for a clear explanation of criteria and procedures for data collection we encourage to consult the original publication that produced the data we re-analyzed
Population characteristics	We collected 16S amplicon (16S) and shotgun metagenomics (SMG) datasets related to case-control studies that compared the composition of the gut microbiome between PD and controls. We include all studies irrespectively of the inclusion/exclusion criteria used, the typology and severity of PD, and the country of origin.
Recruitment	For a clear explanation of criteria and procedures for recruitment and data collection we encourage to consult the original publication that produced the data we re-analyzed.
Ethics oversight	For a clear explanation of criteria and procedures for recruitment and data collection we encourage to consult the original publication that produced the data we re-analyzed.

Note that full information on the approval of the study protocol must also be provided in the manuscript.

Field-specific reporting

Please select the one below that is the best fit for your research. If you are not sure, read the appropriate sections before making your selection.

☒ Life sciences ☐ Behavioural & social sciences ☐ Ecological, evolutionary & environmental sciences

For a reference copy of the document with all sections, see nature.com/documents/nr-reporting-summary-flat.pdf

Life sciences study design

All studies must disclose on these points even when the disclosure is negative.

Sample size	We collected 22 datasets, of which 16 and 6 studies profiled the gut microbiome using 16S and SMG sequencing, respectively. We processed a total of 4,489 samples obtained from case-control studies that profiled the faecal microbiome of PD patients and controls using 16S amplicon (3,165 samples) and shotgun metagenomics sequencing (1,324 samples). Moreover, we re-analysed 10 additional dataset related to multiple sclerosis and Alzheimer's disease. To capture the high variability observed in the gut microbiome across countries and population we aimed at collecting as many datasets as possible. To the best of our knowledge, to date, our meta-analysis is the largest ever performed on

	the gut microbiome of Parkinson's disease patients.
Data exclusions	We excluded all studies that profiled <30 samples, did not make raw data available, or for which it was not possible to assign the samples to patients or controls due to the lack of basic metadata. Also, 16S samples with <2000 reads were discarded.
Replication	We re-analysed 22 datasets in a standardized way starting from taxonomix profiling to statistical analyses. We harmonized the data derived from all these studies and discuss in the manuscript where our main results reproduce previous findings or are inconsistent with these.
Randomization	Not applicable for this study
Blinding	Not applicable for this study

Reporting for specific materials, systems and methods

We require information from authors about some types of materials, experimental systems and methods used in many studies. Here, indicate whether each material, system or method listed is relevant to your study. If you are not sure if a list item applies to your research, read the appropriate section before selecting a response.

Materials & experimental systems

n/a	Involved in the study
☒	☐ Antibodies
☒	☐ Eukaryotic cell lines
☒	☐ Palaeontology and archaeology
☒	☐ Animals and other organisms
☒	☐ Clinical data
☒	☐ Dual use research of concern
☒	☐ Plants

Methods

n/a	Involved in the study
☒	☐ ChIP-seq
☒	☐ Flow cytometry
☒	☐ MRI-based neuroimaging

Plants

Seed stocks	*Report on the source of all seed stocks or other plant material used. If applicable, state the seed stock centre and catalogue number. If plant specimens were collected from the field, describe the collection location, date and sampling procedures.*
Novel plant genotypes	*Describe the methods by which all novel plant genotypes were produced. This includes those generated by transgenic approaches, gene editing, chemical/radiation-based mutagenesis and hybridization. For transgenic lines, describe the transformation method, the number of independent lines analyzed and the generation upon which experiments were performed. For gene-edited lines, describe the editor used, the endogenous sequence targeted for editing, the targeting guide RNA sequence (if applicable) and how the editor was applied.*
Authentication	*Describe any authentication procedures for each seed stock used or novel genotype generated. Describe any experiments used to assess the effect of a mutation and, where applicable, how potential secondary effects (e.g. second site T-DNA insertions, mosiacism, off-target gene editing) were examined.*
